# Assessing insect biodiversity with automatic light traps in Brazil: Pearls and pitfalls of metabarcoding samples in preservative ethanol

**DOI:** 10.1002/ece3.6042

**Published:** 2020-02-25

**Authors:** Mauricio M. Zenker, Alexandre Specht, Vera G. Fonseca

**Affiliations:** ^1^ Zoological Research Museum Alexander Koenig Bonn Germany; ^2^ Embrapa Cerrados Brasilia Brazil; ^3^ Present address: Rua Eulo Maroni São Paulo Brazil; ^4^ Present address: Embrapa Cerrados Planaltina Federal District Brazil; ^5^ Present address: Centre for Environment Fisheries and Aquaculture Science (Cefas) Weymouth UK

**Keywords:** Brazilian biomes, eDNA metabarcoding, insect biodiversity, light traps

## Abstract

Automated species identification based on data produced with metabarcoding offers an alternative for assessing biodiversity of bulk insect samples obtained with traps. We used a standard two‐step PCR approach to amplify a 313 bp fragment of the barcoding region of the mitochondrial COI gene. The PCR products were sequenced on an Illumina MiSeq platform, and the OTUs production and taxonomic identifications were performed with a customized pipeline and database. The DNA used in the PCR procedures was extracted directly from the preservative ethanol of bulk insect samples obtained with automatic light traps in 12 sampling areas located in different biomes of Brazil, during wet and dry seasons. Agricultural field and forest edge habitats were collected for all sampling areas. A total of 119 insect OTUs and nine additional OTUs assigned to other arthropod taxa were obtained at a ≥97% sequence similarity level. The alpha and beta diversity analyses comparing biomes, habitats, and seasons were mostly inconclusive, except for a significant difference in beta diversity between biomes. In this study, we were able to metabarcode and HTS adult insects from their preservative medium. Notwithstanding, our results underrepresent the true magnitude of insect diversity expected from samples obtained with automatic light traps in Brazil. Although biological and technical factors might have impacted our results, measures to optimize and standardize eDNA HTS should be in place to improve taxonomic coverage of samples of unknown diversity and stored in suboptimal conditions, which is the case of most eDNA samples.

## Graphical abstract

In this study, we have used a standard metabarcoding methodology to assess the biodiversity of insects in Brazil from their preservative medium. Our results suggest that obtaining adult insect DNA directly from preservative ethanol can pose a challenge to the use of metabacroding in insect monitoring surveys using automatic light traps, although valuable biodiversity data may be obtained using this approach as well. We here report, for the first time, the insect biodiversity patterns found in Brazil using a metabarcoding approach and discuss the use of preservative ethanol in such approach.

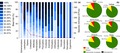

## 1 INTRODUCTION

Insects are arguably the most ubiquitous component of animal biodiversity in the terrestrial ecosystem, but despite their central ecological role in natural and subnatural habitats and their relevance to areas such as agriculture, public health, and biotechnology, we can only estimate how many species of insects are there on earth (Magurran & McGill, 2011; Mora, Tittensor, Adl, Simpon, & Worm, 2011). It is a fact that biodiversity in the temperate parts of the world, mostly in developed countries, is relatively well known compared with tropical regions, and thus, the current numbers of global insect diversity are generally biased toward temperate species (Titley, Snaddon, & Turner, 2017). While in tropical regions, the extremely high diversity of insects and the lack of specialized taxonomists are the main constraints to producing comprehensive species lists of insects (Paknia, Sh, & Koch, 2015), either in temperate or in tropical regions cryptic species seem to be the major problem since these require expert taxonomic assistance (Pfenninger & Schwenk, 2007). Traditional taxonomic approaches of insect inventories in which a multidisciplinary team of taxonomists has to be assembled to identify thousands of morphospecies are prohibitive because of the high cost and manpower required, especially considering tropical areas (e.g., Basset et al., 2012), and thus, alternative approaches must be used to improve our knowledge on insect diversity.

As part of the ongoing advancements in biology, molecular biology, and bioinformatics, several tools are currently used to produce relatively inexpensive automated species identification that can potentially replace or at least complement traditional biodiversity assessment of insects based on morphology (Gibson et al., 2014; Yu et al., 2012). Such approaches include metabarcoding insects from bulk environmental samples (Ji et al., 2013; Kocher et al., 2016) or using insect mock communities (Yu et al., 2012). To deliver taxonomic information, these methods rely strongly on curated genomic data and its assigned taxonomy must be available in online repositories. To enable the assembly of a large database that can be used worldwide, an ideal automated species identification method must use a single easily amplifiable genomic region which is capable to show speciation events (Taberlet, Coissac, Pompanon, Brochmann, & Willerslev, 2012). In addition, for a suitable gene region, such method must also ensure that the taxonomic information assigned to the sequences in the repository can be traced back to museum vouchers identified by a taxonomist (Hebert, Cywinska, Bal, & deWaard, 2003). Among several candidate genes, the barcode region of the mitochondrial COI gene was the region of choice for most metazoan groups. Additionally, since early 2000s, a high number of barcode sequences have been deposited in the BOLD Systems online database and GenBank (Ratnasingham & Hebert, 2007, 2013). Although other barcode gene regions are available for different taxa (e.g., ITS for fungi and rbcL for plants), COI is particularly effective in distinguishing animals at the species level, and a higher number of COI barcode sequences are available in online repositories than of any other gene (Ratnasingham & Hebert, 2013). Therefore, despite some drawbacks (e.g., Stoeckle & Thaler, 2014), the COI gene has been the main choice for automated species identifications of insects.

High throughput sequencing has been used to assess biodiversity from sampling environmental DNA (eDNA) and/or taxon DNA (bulk organisms) using polymerase chain reaction (PCR) to amplify a gene (s) region(s), allowing the identification of a broad spectrum of taxa (aka metabarcoding) (e.g., Fonseca, 2018; Taberlet, Bonin, Zinger, & Coissac, 2018). This method has been used to assess biodiversity of a variety of prokaryotes and eukaryotes, from Archaea/Bacteria and Fungi (Bahram, Anslan, Hildebrand, Bork, & Tedersoo, 2018; Hartmann, Frey, Mayer, Mäder, & Widmer, 2015) to plants and vertebrates (Fahner, Shokralla, Baird, & Hajibabaei, 2016; Hänfling et al., 2016), and in the most distinct ecosystems such as in deep‐sea trenches (Yu, Liang, Niu, & Wang, 2017), the Antarctic (Fonseca et al., 2017), tropical forests (Mahé et al., 2017; Porazinska, Giblin‐Davis, Powers, & Thomas, 2012; Ritter, Häggqvist, et al., 2019b; Ritter, Zizka, et al., 2019a; Ritter et al., 2018) and vertebrate gut content (Vesterinen, Lilley, Laine, & Wahlberg, 2013), among many others. Perhaps, the greatest advantage of metabarcoding eDNA over most methods of field‐based community data collection is that it can be done in a noninvasive fashion (Creer et al., 2016; Ritter, Häggqvist, et al., 2019b; Valentini et al., 2016). For example, if one wants to study aquatic or edaphic organisms, barcode sequences can be obtained directly from water and soil samples since these organisms release DNA molecules that are solubilized in the aqueous phase or absorbed on the surface of different types of organic and mineral particles (Levy‐Booth et al., 2007; Pietramellara et al., 2009). Therefore, metabarcoding eDNA allows the possibility to reduce the sampling effort and costs of biodiversity assessments while increasing the number of detected taxa without affecting local populations (Borrell, Miralles, Do Huu, Mohammed‐Geba, & Garcia‐Vazquez, 2017; Carew, Kellar, Pettigrove, & Hoffmann, 2018).

Different approaches have been used to assess arthropod and especially insect biodiversity with metabarcoding. In water and soil dwelling insects, the DNA can be extracted from water and soil samples, respectively (Taberlet et al., 2018), but the immature stages of many adult flying insects occupy very particular microhabitats such as under tree bark, in tree holes and tree epiphytes, in vertebrate body cavities or in different structures associated to their skin, inside animal and plant tissue, etc. (Borror, Triplehorn, & Johnson, 1989), and thus, it is very difficult to find their DNA in water and soil samples. Alternatively, insect DNA has been metabarcoded from tissue samples and/or whole specimens obtained from traps that are highly effective for many flying insects, although in some cases, it leads to sample destruction and thus loss of vouchers for species identification (e.g., Gibson et al., 2014; Ji et al., 2013; Matos‐Maraví et al., 2018; Ritter, Häggqvist, et al., 2019b). Recent studies showed that insect DNA can also be obtained from their preservative medium allowing the possibility of using the ethanol of insect samples obtained with traps in metabarcoding studies (Hajibabaei, Spall, Shokralla, & Konynenburg, 2012; Shokralla, Singer, & Hajibabaei, 2010).

Brazil is one of the most important countries in the world from a biodiversity perspective (Myers, Mittermeier, Mittermeier, Fonseca, & Kent, 2000). However, because of the extremely high diversity and the difficulty in having access to remote areas of the country, diversity assessments of insects are very difficult to implement at a national scale. Although the number of insect taxonomists in Brazil has increased in the last decades (Rafael, Melo, Carvalho, Casari, & Constantino, 2012), most species inventories/assessments are restricted to areas close to universities and research facilities and/or to a particular taxon like order or family (Lewinsohn, Freitas, & Prado, 2005). The crop damage caused by a caterpillar species recently introduced in Brazil (Sosa‐Gomez et al., 2016) has prompted a national‐scale monitoring program funded by the Ministry of Agriculture, and a network of automatic light traps was established in 12 sampling areas throughout Brazil with the aim of recording abundance of *Helicoverpa armigera* (A. Specht, 2015, personal communication). The trap used in this monitoring program attracts a variety of insects (Kato et al., 1995), which are killed and stored in containers filled with ethanol, offering a great opportunity of using the metabarcoding approach to noninvasively assess insect diversity in a large geographical scale.

In this study, we used the preservative ethanol of insect samples obtained with light traps in 12 sampling areas in Brazil to produce a species list and compare alpha and beta diversity between forest edge and agricultural fields, wet and dry seasons and the main biomes of the country. We here report, for the first time, that the preservative ethanol of adult insect samples obtained with automatic light traps can be successfully used in a metabarcoding study. However, our results also suggest that the preservative ethanol must be stored in proper conditions to avoid insect DNA degradation and to increase PCR success. In addition to reporting our results and discussing the issues we found, we also suggest alternatives on how to produce a metabarcoding study with preservative ethanol of insect samples.

## 2 MATERIAL AND METHODS

### 2.1 Field sampling, sample storage, and ethanol collection

**Figure 1:**
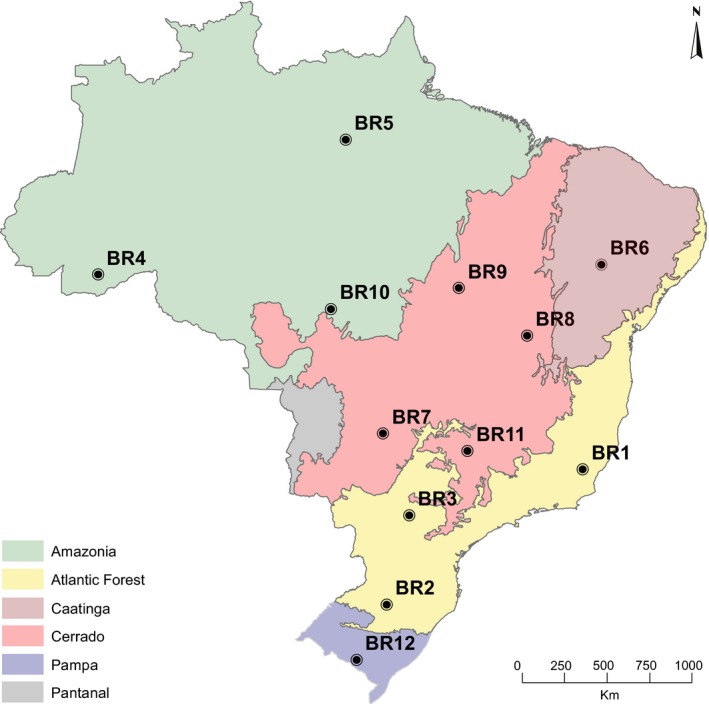
Map with the location of 12 sampling areas included in this study. The biomes of Brazil are identified with different colors

The DNA sequences used in this study were obtained from samples collected during a pest surveillance program in Brazil between May 2016 and February 2017. A total of 12 sampling areas covering all regions and the main biomes of Brazil (Figure 1) were sampled monthly with automatic light traps during wet and dry seasons. One automatic light trap (Zenker et al., 2015) operated from dusk to dawn every new moon period in both agricultural and natural habitats in the same area for five nights in a row, totaling 10 samples per sampling area/month.

To allow transportation of samples by airplane from the sampling areas to the research facilities of Embrapa Cerrados, in central Brazil, the ethanol used to collect the insects had to be drained. Fresh 98% ethanol was added shortly after the arrival at the laboratory, and samples were stored at room temperature for six months before the ethanol was used for downstream analyses. During this period of time, DNA is expected to release from the specimens into the preservative ethanol (Shokralla et al., 2010). Each one of the 112 ethanol samples used in this study comprised a pool of five samples obtained monthly in each habitat/sampling area/season, although in some cases samples obtained in different months in the same season were used for the same habitat/sampling area (Table S1). A disposable pipette was used to collect 10 ml of ethanol from each of the five samples, and the aliquots were pooled into a 50‐ml sterile Falcon tube identified with the name of the sampling area, type of habitat, and season. All samples were stored at room temperature for a period of time varying from 7 to 15 months until DNA extraction.

### 2.2 DNA extraction, amplification, and sequencing

Previously, to the extraction procedure, the samples were manually swirled for 5 s to avoid concentration of DNA in the bottom of the container and to increase the chances of amplifying DNA from all arthropods contained in the sample. Additionally, to increase sample representativeness and coverage, triplicates of 10 ml subsamples (i.e., pseudoreplicates) were aliquoted from each sample, totaling 336 subsamples of the 112 samples. The aliquoted subsamples from the same sample were pooled into a 50‐mL Falcon tube with a sieve attached to its opening. The sieve was changed between samples and was equipped with a 0.45‐µm filter membrane used to avoid the presence of insect fragments during the extraction procedure, and thus the over‐representation of a particular taxon. All subsamples were dried at 56°C in an incubator until the ethanol evaporated. Genomic DNA was extracted using DNeasy Blood and Tissue Kit (Qiagen) according to manufacturer's instructions. A negative control was included in all DNA extraction batches.

All DNA subsamples were PCR‐amplified using a 313 bp long region of the cytochrome c oxidase subunit I mitochondrial gene (COI), with forward (mlCOIintF: GGWACWGGWTGAACWGTWTAYCCYCC) and reverse (jgHCO: TGRTTYTTTGGTCACCCTGAAGTTTA) primers developed by Leray et al. (2013). We choose to amplify a COI region because of the large amount of insect taxonomic information available for this gene in the online repositories (see Deagle, Jarman, Coissac, Pompanon, & Taberlet, 2014). Although there are different pairs of primers available to amplify different regions within the COI gene, tests previously performed in our laboratory with a set of COI primers suggested that the pair of primers deployed in this study is more efficient in amplifying DNA form preservative ethanol samples (data not shown). Subsequent eDNA HTS libraries were performed similarly to Fonseca and Lallias (2016) using a two‐step PCR amplification. First, the targeted region was amplified with specific forward and reverse primers tailed on their 5′‐end by Illumina sequencing priming sites. The first PCR was carried out in 25 µl reaction volumes containing 12.5 µl of Q5® Hot‐start High‐Fidelity 2X Master Mix, 0.8 µl of BSA, 2.1 µl of PCR grade water, 0.8 µl of forward and reverse primers, and 8 µl of DNA using a thermocycling profile of 98°C for 2 min, 25 cycles of 98°C for 40 s, 45°C for 40 s, 72°C for 30 s; and final extension at 72°C for 3 min. PCR1 products were then cleaned with ExoSAP‐ITTM to remove excess primers and unincorporated nucleotides. Second, PCR1 products were reamplified to attach the index and Illumina adapters (P5 and P7). These indexes were used to identify the different subsamples and to increase the number of subsamples analyzed simultaneously within the same sequencing lane (Fonseca & Lallias, 2016). The second PCR was carried out in 25.9 µl reaction volumes containing 12.5 µl of Master Mix, 1.0 µl of BSA, 2 µl of water, 1.2 µl of forward and reverse primers, and 8 µl of PCR1 product using a thermocycling profile of 98°C for 2 min, 20 cycles of 98°C for 40 s, 55°C for 30 s, 72°C for 30 s; and final extension at 72°C for 3 min. To test for possible cross‐contamination during PCR procedures, three negative controls were included in the PCRs and visualized on a 2% agarose gel and sequenced together with the subsamples. All PCR2 products were visualized and posteriorly purified in a 2% agarose gel (QIAquick Gel Extraction Kit, Qiagen) and quantified using the Agilent Bioanalyser (Promega). Equimolar amounts of the amplicon tag‐generated libraries (3 ng/µl) were pooled and sequenced on a Miseq platform using the v3 Illumina chemistry following the 2 × 300 bp paired‐end sequencing protocol at the Centre for Genomic Facilities at the University of Liverpool, UK.

### 2.3 High throughput sequencing data analyses

The initial quality control was carried out at the sequencing center. The raw FASTQ files were trimmed for the presence of Illumina adapter sequences using Cutadapt 1.2.1 (Martin, 2011); the option ‐o 3 was used to remove any reads which match the adapter sequence for 3 bp or more. To avoid incorrectly called bases, the reads were further trimmed using Sickle version 1.33 (Joshi & Fass, 2011) with a minimum window quality score of 20; the reads shorter than 20 bp after trimming were removed.

A pipeline of several command line programs (Appendix S1) was run in a Linux platform to manage and filter the high number of sequences and to obtain OTUs and their respective taxonomic information. The QIIME 1.9.1 (Caporaso et al., 2010) join_paired_ends.py command was used to align both the forward and reverse reads based on their 3′‐end and reconstitute the full‐length sequences. The amplicons were then assigned to its initial name (i.e., subsample name) using QIIME split_libraries_fastq.py command according to P5 and P7 tags added during the second PCR, and Cutadap was used to separate the metabarcode sequence from the primers sequences. The program vsearch (Rognes, Flouri, Nichols, Quince, & Mahé, 2016) was used in many steps. It was used to sort the sequences by length and discard either sequences shorter than 250 bp or longer than 500 bp. To reduce the number of sequences in the data set and the computational time of the analyses, vsearch was used to dereplicate the sequences and store the number of redundant sequences removed from each one of the dereplicated sequences in its header. Additionally, the sequences were sorted in the order of decreasing abundance (number of copies in the dereplicated sequences) and then checked for the presence of chimeras using abundance and reference database approaches (Rognes et al., 2016). The reference file used in the chimera detection steps and to assign taxonomy to the OTUs obtained in further steps was downloaded from the GenBank database. Vsearch was also used to cluster the chimera free sequences into OTUs (95% threshold) and to sort them in the order of decreasing abundance. The Python script/xxx/fasta_number.py was used to rename the OTUs with the initials “OTU_” and a number (e.g., OTU_1, OTU_2, etc.); the file with the renamed OTUs was then used as a database and the file obtained in the dereplication step as an input to map back the number of sequences in each renamed OTU and store this information in a UC file using vsearch. The Python script/xxx/uc2otutab_mod.py was used to convert the UC file to a text file, so a representative sequence of each OTU could be screened against the database downloaded from the GenBank using the tools available at https://blast.ncbi.nlm.nih.gov/Blast.cgi. A similarity level of 90% was used in the BLAST procedure. The BLASTn program was used with the following parameters: max_target_seqs 1, ‐max_hsps 1, and num_threads 8 (see Appendix S1 for the complete script). Finally, a costume made Perl script was used to create a summary table with OTU name, taxonomy information assigned from the database, GenBank identifier, percentage of similarity (varying from 90% to 100%), number of sequences obtained in each subsample, and the representative sequence used in the BLAST step.

### 2.4 Diversity data analyses

The summary table was used to perform alpha and beta diversity analysis and to graphically describe the large amount of data obtained. Sequence similarity level used to identify OTUs employed in many barcoding and metabarcoding studies of insects depends ideally on the taxa being analyzed, but in general, it varies from 95% to 99% (e.g., Gibson et al., 2014; Zenker et al., 2016). Although the OTUs taxonomic identifications obtained with a sequence similarity interval ≥90% are reported, all statistical analyses were performed only with OTUs identified with a sequence similarity ≥97% and ≥98%. In order to make ecological comparisons between habitats, the raw data were normalized to the same number of reads per sample site. All diversity analyses performed with the main data sets were done using both nonnormalized and normalized data sets, and all results are shown.

To statistically compare alpha diversity between biomes, habitats, and season, the rarefaction and extrapolation sampling curves of Hill numbers for incidence data were used (Chao et al., 2014; Chao & Jost, 2012). Different data sets were analyzed, including those with OTUs identified at 97% and 98% similarity levels, with and without singletons, doubletons, and tripletons (herein referred as SDTs), and normalized data sets. These analyses were done using the program iNext (Hsieh, Ma, & Chao, 2013 available from http://chao.stat.nthu.edu.tw/inext/) configured at 40 knots; 95% confidence intervals were generated by the bootstrap procedure (300 bootstraps). To analyze differences in community composition, we used nonmetric multidimensional scaling (NMDS) and analysis of similarities (ANOSIM), based on Bray–Curtis dissimilarities considering incidence data. These analyses were run in R (R Development Core Team, 2013), using the package “vegan” (Oksanen et al., 2013).

## 3 RESULTS

### 3.1 OTUs taxonomic assignment

**Figure 2:**
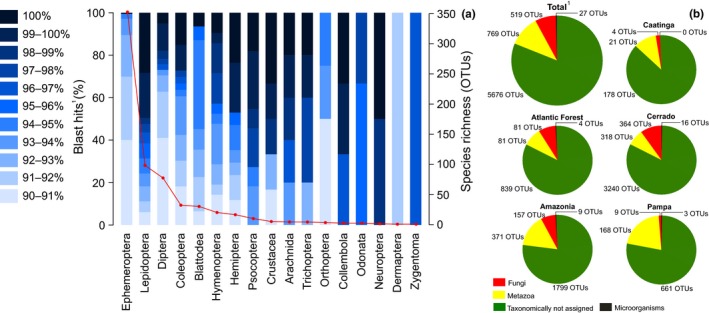
Summary results for OTU clustering and taxonomic assignments obtained from preservative ethanol samples collected in 12 sampling areas in Brazil with automatic light traps. (a) Different levels of species taxonomic assignment for 13 insect orders and four additional arthropod taxa are provided in bar charts with different degrees of blue. The red dots represent the number of OTUs obtained for each taxa. (b) Proportion and number of taxonomically unassigned OTUs and OTUs included in higher taxa for total data set and for five biomes in Brazil are provided in pie charts, ^1^including negative controls

A total of 69 subsamples representing 36 samples out of 112 were PCR‐amplified successfully using the COI gene and considered suitable for sequencing with an average concentration of 6.05 ng/µl. This represents 32.14% of the total number of samples and 20.53% of the subsamples available for this study. The number of samples was higher in Cerrado, followed by Amazonia, Atlantic Forest, Pampa, and Caatinga; and the number of subsamples was higher in Cerrado, Atlantic Forest, Amazonia, Pampa, and Caatinga (Table 1). All subsamples were successfully sequenced, and after chimera removal, a total of 8,097,062 sequences were clustered into 6,899 OTUs (Table 1); the sequences were deposited at the GenBank/EMBL/DDBJ short read archive, study number PRJNA599423. Additionally, a total of 92 OTUs were obtained from 25 negative controls in which 52,527 sequences were present (Table S1). In total, only 18.81% of the OTUs were assigned to a species name with a ≥90% similarity level (BLAST match), and a similar result was obtained for individual biomes (Figure 2). More than half of the OTUs assigned to species level were metazoans, although a high number of OTUs were assigned to Fungi and a few to other higher taxa (Table S1). Metazoans were more abundant in Amazonia, Caatinga, and Pampa; fungi were more abundant in Cerrado; and metazoans and fungi were equally abundant in the Atlantic Forest (Figure 2). A total of 161 and 146 out of 769 OTUs assigned to Metazoa were identified at ≥97% and ≥98% similarity levels, respectively. Additionally, 20 OTUs found in the negative controls were identified at a ≥97% similarity level (19 OTUs at ≥98%), although only five of these were found exclusively in the negative controls (i.e., three microorganisms and two insects).

**Table 1:** Total number of sequences and OTUs, and number of OTUs assigned to Arthropoda obtained from preservative ethanol used in automatic light traps in a pest monitoring program in Brazil during 2016 and 2017

Sampling sites	Biomes	Samples	Subsamples	Sequences^a^	OTUs^b^	OTUs assigned to Arthropoda^b^	Arthropod OTUs (≥97% similarity level)
BR01	Atl. Forest	1	1	179,161	32	2	1
BR02	Atl. Forest	2	3	419,399	366	7	1
BR03	Atl. Forest	4	9	769,489	717	57	10
BR04	Amazon	5	6	817,129	1,180	112	23
BR05	Amazon	3	6	772,403	1,381	254	23
BR06	Caatinga	1	2	469,645	113	19	9
BR07	Cerrado	5	12	1,551,955	2,081	142	56
BR08	Cerrado	3	5	445,456	606	60	9
BR09	Cerrado	3	6	575,704	565	28	10
BR10	Cerrado	4	8	1,092,232	1,080	55	27
BR11	Cerrado	2	3	203,359	382	13	8
BR12	Pampa	3	8	801,130	713	163	3
Total^c^		36	69	8,097,062	6,899	669	128

a Total number of sequences obtained after the chimera detection steps.

b OTUs identified at a ≥90% similarity level.

c excluding negative controls (see text for details).

Approximately 87% of the total animal OTUs were assigned to 14 insect orders and three additional arthropod taxa (Table 1, Figure 2). The number of OTUs assigned to Ephemeroptera was abnormally high because a total 350 OTUs hit the same sequence in the database (GenBank ID: KX039561.1) with similarity levels ranging from 90% to 94.69%. Additional 27 OTUs ranging from 90.71% to 95.99% and one OTU with 100% similarity hit a single sequence in the database (GenBank ID: KM577141.1) and markedly increased the number of Blattodea OTUs (Table S1). The Lepidoptera was the second insect order with the highest number of OTUs (*N* = 99) followed by Diptera (*N* = 78) and Coleoptera (*N* = 33); Hymenoptera, Hemiptera, and Psocoptera were less abundant, with 21, 17, and 11 OTUs, respectively; relative abundances were very low for the remaining arthropod taxa, between one to six OTUs (Figure 2). The remaining 13% of animal OTUs were assigned to six different phyla and a taxonomically unidentified sequence (Table 2). Similarly to Ephemeroptera, 22 annelid OTUs were assigned to a single sequence in the database (ranging from 90.2% to 94.89%), and 42 chordate OTUs to four different human sequences ranging from 92.6% to 100%, with only six OTUs higher than 97% (Table S1).

**Table 2:** Number of animal OTUs, except Arthropoda and including negative controls, obtained in the HTPS data analyses. Results should be interpreted cautiously (see Section 4 for details)

Taxon	OTUs ≥90%	OTUs ≥97%
Chordata	62	23
Annelida	26	3
Nemertea	3	3
Nematoda	2	1
Rotifera	2	0
Porifera	1	1
Environmental^a^	1	0

a Sequence identified in the database as “invertebrate environmental sample” (GenBank id: GU070905.1). One OTU assigned to Nematoda and Rotifera at ≥90% similarity level and the “Environmental” OTU were obtained exclusively in the negative controls.

**Figure 3:**
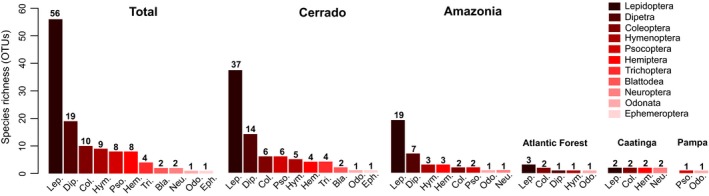
Insect OTUs identified to species level at a ≥97% sequence similarity level (excluding the OTUs obtained in the negative controls) and their respective higher classification. Given are the total number of OTUs and the OTUs obtained in the five different biomes in Brazil

A total of 128 and 114 out of 669 OTUs assigned to Arthropoda were identified at ≥97% and ≥98% similarity levels, respectively (Table S1). The vast majority of OTUs were assigned to insects, although four crustacean, three arachnid, and two springtail OTUs were also found. Additionally, two insect OTUs identified at ≥97%, and one at ≥98%, were detected exclusively in the negative controls. The number of arthropod OTUs identified at a ≥97% similarity level obtained in 12 sampling sites is available in Table 1 and a species list in Table S1. A total of 11 insect orders were obtained at a ≥97% similarity level, although this number varied greatly between biomes. Almost all orders were detected in Cerrado and Amazonia but only two in Pampa, four in Caatinga, and five in Atlantic Forest (Figure 3). The high number of OTUs obtained at a ≥90% similarity level for Ephemeroptera and Blattodea dropped considerably at ≥97% and, as expected, Lepidoptera was the taxa with the highest number of OTUs, followed by Diptera, Coleoptera, and Hymenoptera. There was a difference in the number of OTUs obtained in different biomes, especially when considering the most abundant orders. The Cerrado was the most abundant biome, followed by Amazonia, but the number of OTUs decreased steeply in the remaining biomes (Figure 3).

### 3.2 Alpha and beta diversity analyses

**Figure 4:**
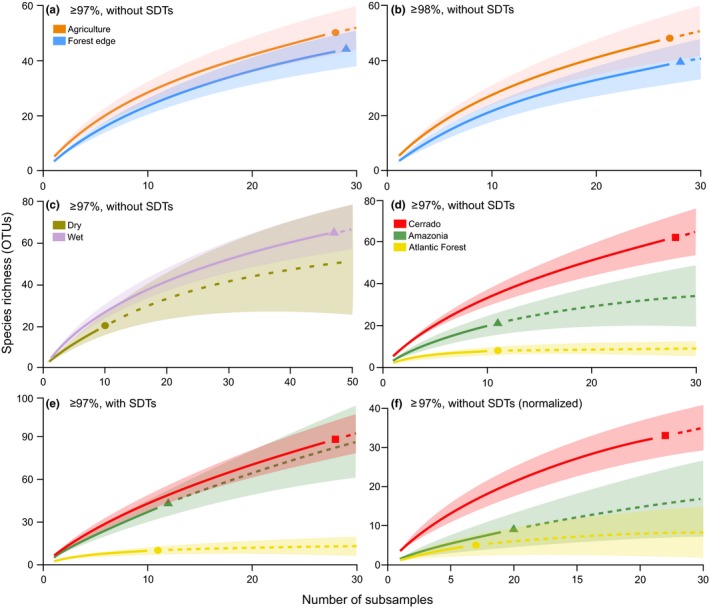
Rarefaction and extrapolation curves with 95% confidence intervals (shaded areas) comparing habitats, biomes, and seasons based on incidence data of arthropod OTUs obtained in 12 sampling areas in Brazil. The OTUs obtained from the negative controls were excluded from the analyses. (a) Data set including OTUs taxonomically assigned at a ≥97% similarity level excluding singletons, doubletons, and tripletons (SDTs); (b) the same data set using a ≥98% similarity level; (c and d) data set using OTUs assigned at a ≥97% similarity level and excluding SDTs; (e) the same data set including SDTs; (f) normalized data set using OTUs assigned at a ≥97% similarity level, and excluding SDTs, obtained after standardizing the number of sequences in the subsamples to 15,840 sequences (see Section 2 for details)

Alpha diversity analyses did not allow a markedly distinction between different habitats and biomes, although a consistent result in which diversity did not vary between dry and wet seasons was obtained in all data sets, including arthropod OTUs identified at ≥97% and ≥98% similarity levels, with and without SDTs, and normalized data sets (Figure 4, Figure S1). The rarefaction and extrapolation curves using OTUs identified at a ≥98% similarity level and excluding SDTs showed that arthropod diversity is significantly different between habitats and higher in the agricultural habitat rather than in the forest edge, although nonsignificant at ≥97% sequence similarity level (Figure 4a,b). The results for the remaining data sets either showed a nonsignificant or significant difference between habitats but in all cases a higher diversity in the agricultural habitat (Figure S1). Alpha diversity was analyzed in Amazonia, Atlantic Forest, and Cerrado data sets, but the remaining biomes had to be excluded from the analyses because of lack of comparability (low number of OTUs and subsamples compare with the other biomes). Three different results were obtained with the different data sets comparing diversity between biomes (Figure 4d–f). Diversity was always higher in Cerrado and significantly different from other biomes in most data sets, although a nonsignificant difference between Cerrado and Amazonia was found in the data sets including SDTs (Figure 4), except for the normalized data set at ≥98% similarity level (Figure S1). Additionally, a nonsignificant difference between diversity in Amazonia and Atlantic Forest was found in the normalized data sets excluding SDTs, although a marginally significant difference was found with the data set obtained at a ≥98% similarity level (Figure S1).

**Figure 5:**
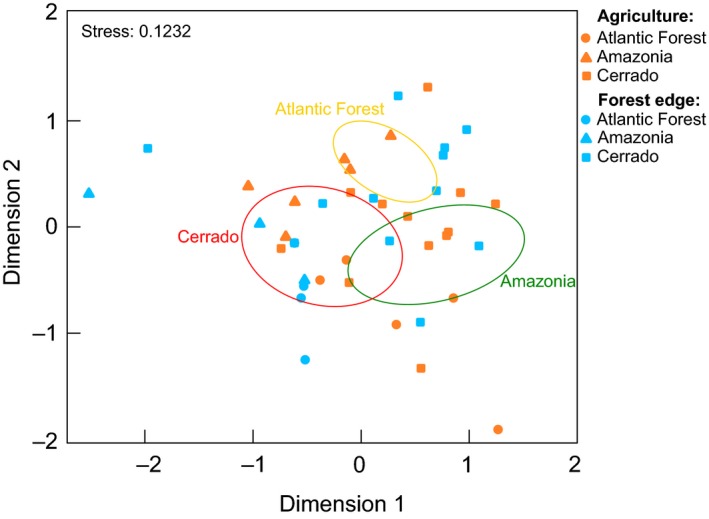
Nonmetric multidimensional scaling (NMDS) of arthropod community similarity recorded in agricultural and forest edge habitats in three different biomes of Brazil. The OTUs used in the NMDS were taxonomically assigned at a ≥97% sequence similarity level. SDTs and OTUs obtained from the negative controls were excluded

The beta diversity analyses showed that the arthropod community composition obtained with OTUs identified at ≥97% and ≥98% similarity levels did not differ significantly between habitats and seasons but rather between Cerrado, Amazonia, and Atlantic Forest (Table 3). These results were similar also when including or excluding SDTs and also when using the normalized data sets (Table 3). The nonmetric multidimensional scaling plot of the taxonomic patterns found for the arthropod communities did not show clear clusters for the different habitats (Figure 5).

**Table 3:** Results of analysis of similarities (ANOSIM) comparing arthropod community composition between different habitats, biomes, and seasons in Brazil. The main data set and a data set in which the number of sequences in the subsamples was standardized (normalized data set) were analyzed including and excluding SDTs (singletons, doubletons, and tripletons). The OTUs obtained from the negative controls were excluded from the analyses. See Section 2 for details

Comparison	Similarity level	Main data set		Normalized data set	
		*R*	*p*	*R*	*p*
Habitats without SDT	≥97%	.03934	.109	.008816	.321
Biomes without SDT	≥97%	.2261	**.002**	.1573	**.006**
Seasons without SDT	≥97%	.1067	.126	.01184	.447
Habitats with SDT	≥97%	.0209	.195	.01703	.222
Biomes with SDT	≥97%	.235	**.002**	.1433	**.006**
Seasons with SDT	≥97%	.08216	.152	.02602	.358
Habitats without SDT	≥98%	.0448	.071	.01098	.328
Biomes without SDT	≥98%	.228	**.001**	.1632	**.004**
Seasons without SDT	≥98%	.06747	.213	−.06337	.744
Habitats with SDT	≥98%	.03416	.094	.01838	.232
Biomes with SDT	≥98%	.2023	**.003**	.1438	**.008**
Seasons with SDT	≥98%	.03919	.304	.03625	.286

**Figure 6:**
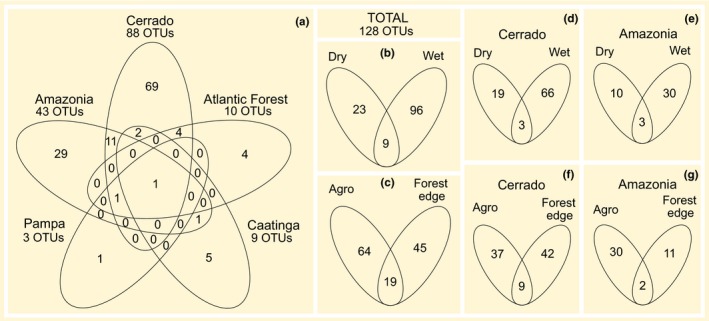
Number of arthropod OTUs shared and unique in the different biomes (a), seasons (b), and habitats (c); and number of arthropod OTUs shared and unique in different seasons in Cerrado (d) and Amazon (e), and in different habitats in Cerrado (f) and Amazon (g). OTUs taxonomically assigned at a ≥97% similarity level. The OTUs obtained from the negative controls were excluded from the analyses

The number of arthropod OTUs obtained in the biomes and the number of arthropod OTUs recorded exclusively in one biome were considerably higher in Cerrado and Amazonia compared with other biomes (Figure 6a). Cerrado shared 11 OTUs with Amazonia, 4 OTUs with Atlantic Forest, and 2 OTUs with Caatinga; Amazonia shared 1 OTU with Caatinga and 1 OTU with Cerrado, Pampa, and Atlantic Forest; 1 OTU was shared between all biomes (Figure 6a). Although the arthropod community composition did not differ significantly between habitats and seasons, a higher number of OTUs were recorded exclusively during the wet season and in the agricultural habitat (Figure 6b,d,e). Similarly, a higher number of OTUs were obtained during the wet season in Cerrado and Amazonia. However, in Cerrado and Amazonia, the number of OTUs was higher in the forest and agriculture habitats, respectively (Figure 6c,f,g).

## 4 DISCUSSION

In this study, we show that DNA metabarcodes representing insect communities can be obtained from the preservative ethanol of bulk adult insect samples. This is important considering that adult insect/arthropod specimens obtained with traps are the physical evidence of biodiversity assessments (Meineke, Davies, Daru, & Davis, 2018) and may represent new species to science that would otherwise be damaged or destroyed if DNA would be extracted from the whole body or tissue samples. Therefore, the ethanol used to preserve adult arthropod/insect samples obtained from traps employed in community ecology field surveys should be regarded as a potential source of data and must not be discarded, but rather carefully preserved (Ritter, Häggqvist, et al., 2019b). Additionally, this study is the first to apply a metabarcoding approach to compare biodiversity patterns of insects in a large geographical area in a neotropical region.

### 4.1 General taxonomic screening and the limited success in obtaining DNA from preservative ethanol samples

Although we have partially successfully used a metabarcoding approach to obtain data on insect communities from Brazil in preservative ethanol, only a low number of samples could be sequenced. The DNA of a total of eight samples and 24 subsamples for each one of the 12 sampling areas was amplified, but PCR amplification products were either absent or insufficient for more than 65% of the samples. Failure in PCR amplification can be due to several causes, from sample preservation and DNA quality to primer design and thermocycler parameters (Taberlet et al., 2018). Although a number of DNA extraction approaches are available for environmental samples such as soil (Dopheide, Xie, Buckley, Drummond, & Newcom, 2019), feces (Rytkönen et al., 2018), and water (Brannock & Halanych, 2015), little attention has been given to preservative ethanol. Excepting, Shokralla et al. (2010) that successfully amplified COI fragments from a single Lepidoptera larva directly from a preservative medium containing 95% ethanol but also mescal solution, followed by first generation sequencing (but see Ritter, Häggqvist, et al., 2019b for a recent method). We have tried Shokralla et al. (2010) method on our samples preserved just in 95% ethanol, but the present methods showed higher DNA and PCR yields at least for the majority of our environmental samples preserved in 95% ethanol. Additionally, in order to augment DNA quality and quantity and further PCR success rate (e.g., decrease PCR inhibitors), other methods were performed. Such as using DNA purification and concentration kits developed for soil and feces samples (PowerSoil® DNA isolation kit and Quick‐DNA™ Fecal/Soil Microbe Kit), nonetheless these did not substantially improve DNA yields (data not shown).

The total number of OTUs evidences that the light traps used in our field survey sampled highly diverse biological communities, nonetheless approximately 82% of the OTUs remained taxonomically unassigned (Table1, Figure 2). OTU taxonomic assignments heavily rely on several factors, from DNA extraction, PCR, and sequencing to the in silico approach used (Fonseca, 2018; Taberlet et al., 2018). A number of errors can occur in the PCR (Fonseca et al., 2012) and sequencing procedures, most notably amplification biases associated with primers (Taberlet et al., 2012). One of the factors that could have caused such high numbers of nonassigned OTUs is the relatively reduced number of COI barcodes from neotropical insect species deposited in GenBank. In fact, in some tropical regions, with a few exceptions (e.g., Janzen & Hallwachs, 2016), insect species are frequently poorly represented, and thus, it is highly likely that many of the unassigned OTUs reported in this study were obtained from species that are not covered in GenBank or in any other database. It is worth to emphasize that automated species identification methods rely on the synergy between taxonomists, molecular biologists, and data scientists. Consequently, it is very important that the efforts endured by the scientific community should be intensified to rapidly populate the databases with valuable data on tropical taxa that can be used to tackle biodiversity crisis (Meyer & Paulay, 2005).

The higher taxonomic assignment of 1,315 OTUs or ca. 18% of the OTUs identified with a ≥90% sequence similarity level showed that more than half were metazoan, but surprisingly, a large number of OTUs were also assigned to fungi species and a few to other microorganisms (Figure 2, Table S1). OTUs taxonomically assigned to microorganisms such as Proteobacteria have been recorded before in a metabarcoding study of arthropods targeting the same COI gene region employed in this study (Gibson et al., 2014). Insects, especially those caught with light traps, are usually flying insects covering highly mobile species that occupy a number of different niches and interact with different organisms (Grimaldi & Engel, 2005). It is thus highly probable that much of the microorganisms detected in this study could have been carried by or attached to the insects caught in the light traps. Moreover, it would have been very difficult or even impossible to avoid the amplification of such biota in highly diverse insect samples using universal primers (Smith et al., 2012). Although the primer pair used in this study was designed to amplify a wide array of metazoans (Leray et al., 2013), recent studies showed that these primers are also capable to amplify fungi DNA (Leray & Knowlton, 2015; Ritter, Häggqvist, et al., 2019b) and, thus, not so surprisingly, fungi OTUs were also found in our samples. However, the high number of OTUs assigned to fungi species and the discrepancies between the number of fungi OTUs obtained in the different biomes may have been caused by the cascade effects of three factors: (a) the logistics in transporting the samples from the sampling areas to the laboratory; (b) the long period of time between the field collection of insect samples and the processing of preservative ethanol samples; and (c) the storage conditions (i.e., room temperature) in which the samples were preserved during this period of time. Even when the ethanol was replaced, such long periods of sample storage from 7 up to 15 months could have changed ethanol concentration due to evaporation and thus increase the chances of DNA degradation and fungi development. Additionally, it is unlikely that a suitable amount of DNA would be available by the time the samples were processed in the laboratory, as shown for water samples (Taberlet et al., 2018). We believe that replacing the ethanol by lysis buffer and if possible grinding these samples would be preferable for better eDNA yields and taxonomic coverage. In the absence of an alternative method to extract eDNA from samples preserved in ethanol from insect light traps, it is advisable to store them under refrigeration immediately after collection and in properly sealed containers to avoid ethanol evaporation (Ritter, Häggqvist, et al., 2019b).

### 4.2 Taxonomic assignment of metazoan OTUs

Sequence similarity cutoffs used in this study (97% and 98%) have been widely used to assign species names to arthropod OTUs using the COI gene (e.g., Gibson et al., 2014; Zenker et al., 2016). Notwithstanding, a recent metabarcoding study of freshwater macroinvertebrates employed different levels of sequence similarity thresholds against public databases to assign taxonomies depending on the taxonomic rank (Elbrecht, Vamos, Meissner, Aroviita, & Leese, 2017). Here, we choose to further report the results obtained from the HTS analyses using a ≥90% similarity level and constraint the taxonomic assignment at the species level to OTUs identified only at ≥97%–98% sequence similarity level.

Three different scenarios may explain the high number of OTUs obtained at a ≥90% similarity level matching a single sequence in GenBank for Ephemeroptera, Blattodea, and Annelida, and four different sequences for Chordata: (a) The OTUs depict high intraspecific variation of the species with the matching sequence in GenBank leading either to nonredundant BLAST assignments and/or assigned OTUs that can split or agglutinate into the same genus or species (Potter et al., 2017); (b) the OTUs represent species phylogenetically close related to the species with the matching sequence in GenBank, but without representative COI barcodes in GenBank; and (c) errors occurred during the sequencing process. A high intraspecific genetic variation in the barcoding region (≥2%–3%) has been reported in Annelida (Kvist, 2016) and Blattodea (Che, Gui, Lo, Ritchie, & Wang, 2017), and also for mayflies in which *Caenis youngi* (Ephemeroptera: Caenidae) had a maximum intraspecific distance ranging from 3.7% to 21.9% (Webb et al., 2012). The high intraspecific distance suggests the presence of cryptic species (e.g., Janzen et al., 2005; Smith et al., 2008), but a number of genetic factors should be considered when establishing species boundaries within mitochondrial sequences. Namely, the presence of intracelular parasites (*Wolbachia*, Xiao et al., 2012), copies of nuclear mitochondrial DNA sequences (NUMTS, Hazkani‐Covo, Zeller, & Martin, 2010), gene introgression in hybrid species (Bachtrog, Hornton, Lark, & Andolfatto, 2006), and the incomplete lineage sorting (Pollard, Iyer, Moses, & Eisen, 2006) are among the factors that might have affected our results and increased the intraspecific distance obtained in the taxa above mentioned. In addition to that, a very small percentage of the sequencing reads (~0.1%, Taberlet et al., 2018) might have been assigned to the wrong sample index during the sequencing process, although a recent study suggests that this is not the main cause of errors in Illumina platforms (Pfeiffer et al., 2018). The same study also reports that the sequencing reads quality control, such as the one employed in this study is capable of correcting such errors.

The high proportional number of moths detected at a ≥97% sequence similarity level, followed by dipterans, coleopterans, and other insects (Figure 3), is consistent to what would be expected from insect samples obtained with automatic light traps (Kato et al., 1995). Additionally, the detection of medically important insect species like *Lutzomyia longipalpis*, the primary vector of visceral leishmaniasis in Latin America, and the invasive pest species *Helicoverpa armigera* highlights the potential of metabarcoding as a biomonitoring tool. The number of insect OTUs varied greatly between samples and subsamples (Table 1), and between biomes (Figure 3). Such discrepancies might suggest taxonomic primer bias, where the number and position of nucleotides are often mismatched between metabarcoding primers pairs and their annealing regions, and thus, it is unlikely that the DNA of all insects would have been amplified equally during PCR amplification (Elbrecht et al., 2017; Leray et al., 2013).

Apart from arthropods, there were other metazoan found in our study at ≥97% sequence similarity level reflecting the sensitivity of the eDNA HTS approach to also detect nontarget species present in the surrounding environment. Some of these species like the common potoo (*Nyctibius griseus*), the little nightjar (*Setopagis parvulus*), and the exotic gekkonid lizard *Hemidactylus maboui* (= *Hemidactylus mercatorius*) are active during the night and prey on flying insects (see Table S1 for a complete list of species). Therefore, considering that these species are commonly found in Brazil, it is highly likely that fragments of feathers, hairs, or any other tissue fragment might have fallen from individuals, which can either travel attached to other animals or eliminated by feces and thus found nearby or inside the sampling pots. Additionally, the same could have also occurred for species of domesticated animals detected in our analysis (i.e., cow and chicken). Conversely, it is highly unlikely that other metazoan species detected in our samples (Table 2, Table S1) would have in fact been found in neotropical habitats, mostly because their occurrence is typically restricted to the Palearctic region or because they are marine species. Such unlikely taxonomic assignments could be associated with lower sequence similarity BLAST thresholds that reflect the closest taxa to the target sequence. Most protocols available for metabarcoding eDNA samples highlight the importance of including negative controls to detect contaminants during DNA extraction and PCR (Taberlet et al., 2018). Despite no PCR amplification product was detected in the negative controls, 13 animal species were identified at a ≥97% similarity level in the negative controls of the extraction batches (Table S1) only eight of them, including *Homo sapiens*, match the species included in the phyla reported in Table 2.

### 4.3 Alpha and beta diversity in neotropical biomes

The discrepant results between the different data sets used to compare alpha and beta diversity (Figure 4, Table 3) can be ascribed to a number of factors. The absence of strong distinct patterns could reflect local environmental features that may cover historical and climate influences on local diversity (Heino, 2009) that were not analyzed in this study. From life‐history strategies to physical parameters such as precipitation levels, air temperature shifts, and even organic compounds found in agricultural and natural areas can greatly impact insect alpha diversity (Vinson & Hawkins, 1998). However, it is highly probable that the cascade effects caused by the sample storage conditions, resulting in poor DNA quality, might have affected downstream HTS efficiency and analyses. Based on the current knowledge of neotropical insect diversity, we would expect a significant difference between wet and dry season (DeVries, Murray, & Lande, 1997; Valente, Zenker, & Teston, 2018), and thus, the nonsignificant difference in alpha diversity between seasons suggests the need to increase the number of biological samples, since in this study, the rarefaction curves were far from reaching a plateau. This further emphasizes how rich and diverse neotropical regions are and the need to conduct biodiversity studies in such habitats. Conversely, the significant difference between communities of different biomes agrees with what would be expected from neotropical insects samples obtained with automatic light traps and sorted with a morphospecies approach (Zenker et al., 2015). Furthermore, the higher number of Lepidoptera OTUs identified at ≥97% similarity level, compared with that of other insect orders, is similar to what would be expected from automatic light traps, as previously found for moths in Brazil (Specht, Teston, Mare, & Corseuil, 2005; Zenker et al., 2015).

Our diversity analyses reiterate the impact of sequence similarity levels (cutoffs) on OTU taxonomic composition (Holovachov, Haenel, Bourlat, & Jondelius, 2017; Potter et al., 2017; Tapolczai et al., 2019) since alpha diversity differed when using different cutoffs. The choice of sequence similarity levels greatly depends upon well‐curated reference databases, marker of choice, targeted taxa, or a combination of all. Unfortunately, in‐house reference databases for less studied or hyper‐diverse regions are scarce or inexistent, but these would increase and improve taxonomic assignments. Either using nuclear or mitochondrial eukaryotic databases, the extent of assignments will differ depending on the target taxa (Holovachov et al., 2017) (e.g., micro or macrofauna) and annotation accuracy if considering rare or new species. Similarly, the inclusion of low abundance OTUs in eukaryotic diversity assessments should occur, if possible, when using stringent sequence similarity cutoffs (e.g., 99%–100% BLAST matches).

### 4.4 Closing remarks

eDNA metabarcoding approaches have been used to assess biodiversity of a number of taxa in several ecosystems in different parts of the world. The results have revealed similar diversity patterns compared with the traditional approach of taxonomic assignment of species based on morphology and in many cases have improved the detectability of species missed with the traditional approach (Creer et al., 2016; Deiner et al., 2017; Taberlet et al., 2012; Valentini et al., 2016). Although metabarcoding methods have been intensively developed in the last ten years, a number of gaps remain demanding further research. In this study, we have successfully used a standard metabarcoding methodology to assess the biodiversity of insects from their preservative medium, but it is clear that our results underrepresent the true magnitude of insect diversity expected from samples obtained with automatic light traps in Brazil. A number of factors might have affected our results (Fonseca, 2018), and we were not able to precisely identify which factors and to what extent they influenced our results. Nonetheless, measures to optimize and standardize eDNA HTS methods are increasing, mainly to improve taxonomic coverage of samples of unknown diversity and stored in suboptimal conditions, such as is the case of most eDNA samples.

## CONFLICT OF INTEREST

None declared.

## AUTHORS' CONTRIBUTIONS

The work presented here was carried out in collaboration between all authors. MMZ, AS, and VGF designed the experiments. VGF devised and supervised the molecular eDNA metabarcoding approach. MMZ undertook the HTS laboratory work, analyzed the data, and carried the biodiversity analyses. MMZ wrote the first draft of the manuscript. AS performed fieldwork. All authors reviewed the manuscript and helped with critical advice and discussion.

## Data Availability

The authors declare that the DNA sequences used in this study were uploaded to the GenBank Sequenced Read Archive, study number PRJNA599423.
