# Supplementary material for: Assessing insect biodiversity with automatic light traps in Brazil: Pearls and pitfalls of metabarcoding samples in preservative ethanol
Source: Ecol Evol. 2020 Feb 25;10(5):2352–66. doi: 10.1002/ece3.6042 (PMC7069332; doi:10.1002/ece3.6042)

Appendix online IV. Rarefaction/extrapolation curves with 95% confidence intervals (shaded areas) obtained with different data sets comparing habitats seasons, and biomes

97% similarity level

98% similarity level

Without sing. doub. and tripl. | With sing. doub. and tripl. | Without sing. doub. and tripl. | With sing. doub. and tripl.

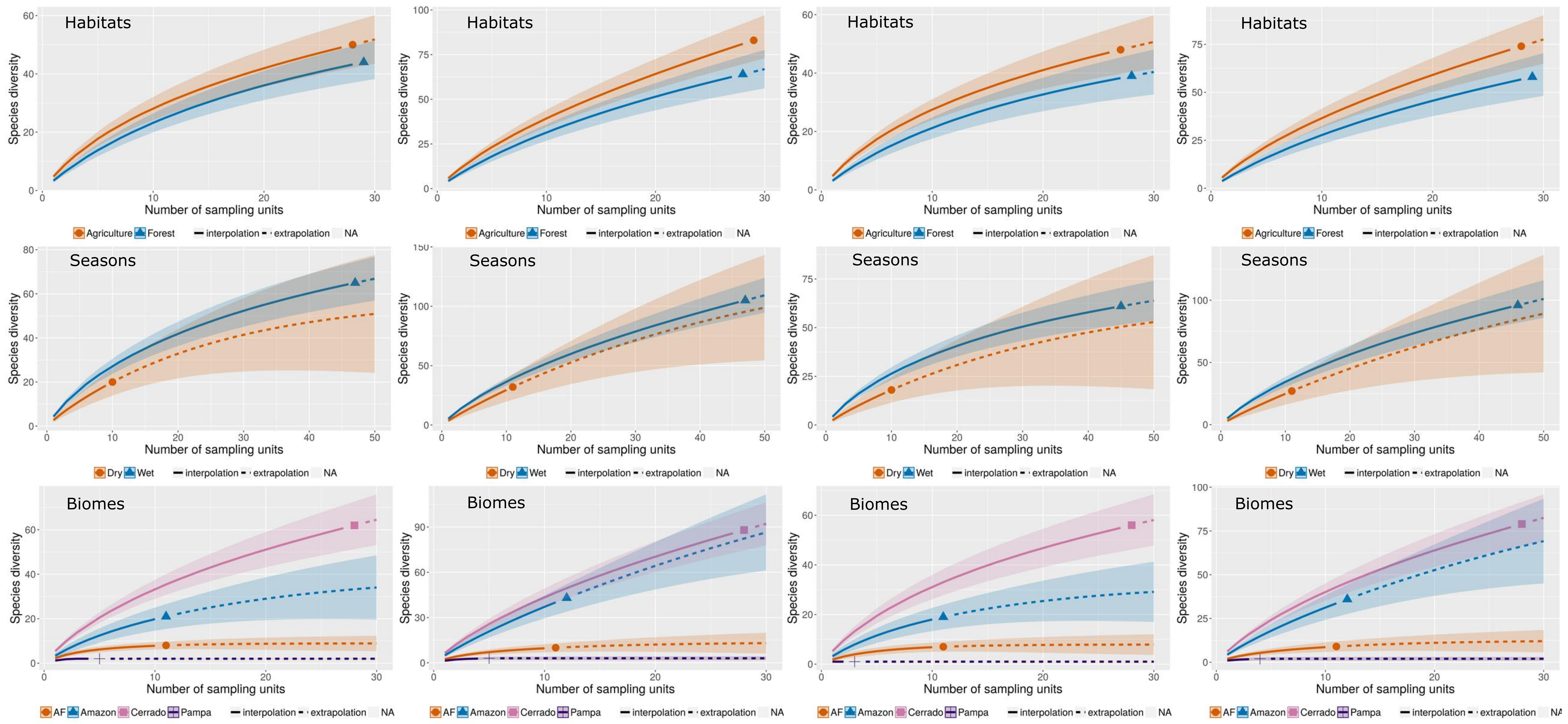

NORMALIZED
n = 15840

97% similarity level

98% similarity level

Without sing. doub. and tripl. | With sing. doub. and tripl. | Without sing. doub. and tripl. | With sing. doub. and tripl.

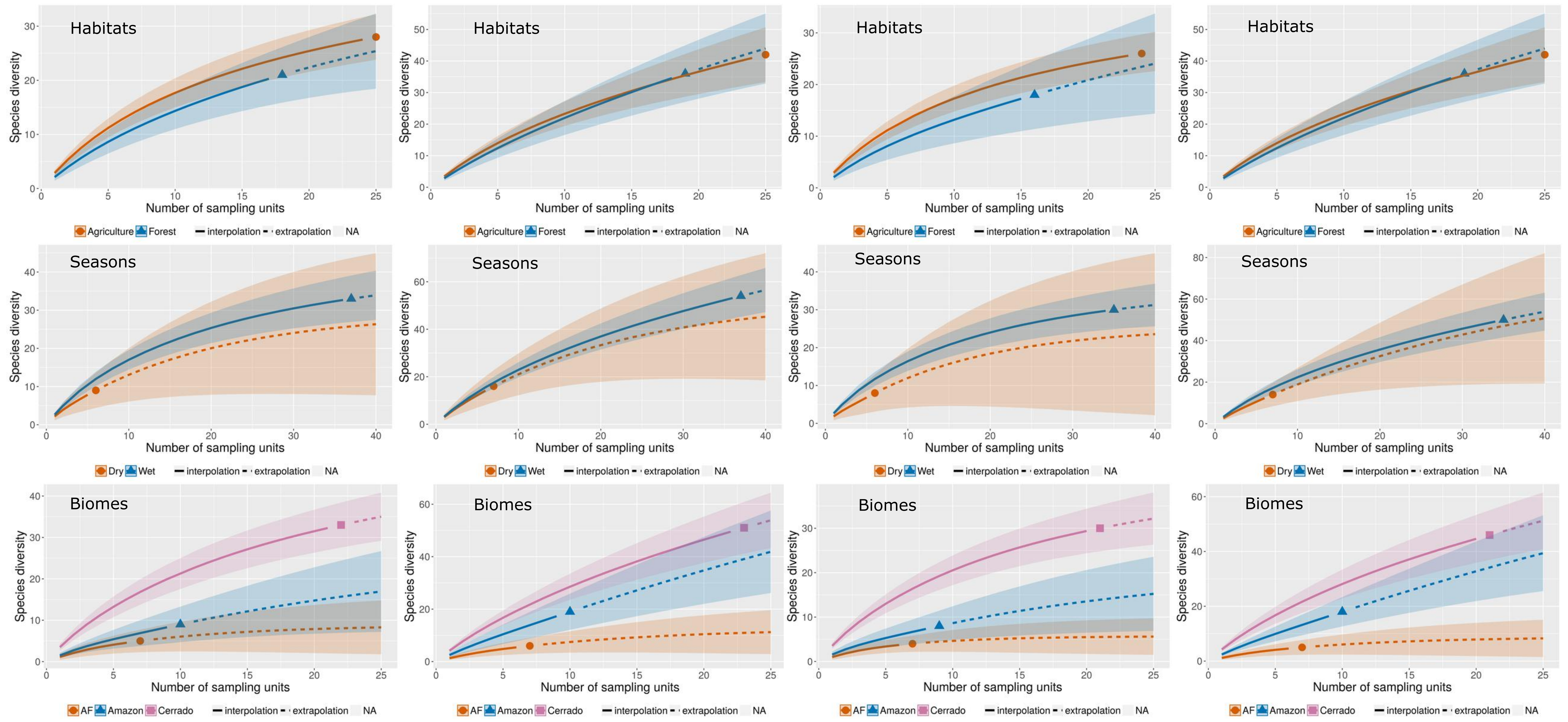

Supplement: Supplementary file 1 [file ECE3-10-2352-s001.pdf]
